# Induction of IFN-β through TLR-3– and RIG-I–Mediated Signaling Pathways in Canine Respiratory Epithelial Cells Infected with H3N2 Canine Influenza Virus

**DOI:** 10.4014/jmb.2010.10047

**Published:** 2021-05-27

**Authors:** Woo-Jung Park, Sang-Hoon Han, Dong-Hwi Kim, Young-Jo Song, Joong-Bok Lee, Seung-Yong Park, Chang-Seon Song, Sang-Won Lee, In-Soo Choi

**Affiliations:** Department of Infectious Diseases, College of Veterinary Medicine, Konkuk University, Seoul 05029, Republic of Korea

**Keywords:** Canine influenza virus, TLR3, RIG-I, IFN-β, MX1

## Abstract

Canine influenza virus (CIV) induces acute respiratory disease in dogs. In this study, we aimed to determine the signaling pathways leading to the induction of IFN-β in a canine respiratory epithelial cell line (KU-CBE) infected with the H3N2 subtype of CIV. Small interfering RNAs (siRNAs) specific to pattern recognition receptors (PRRs) and transcription factors were used to block the IFN-β induction signals in H3N2 CIV-infected KU-CBE cells. Among the PRRs, only the TLR3 and RIG-I expression levels significantly (*p* < 0.001) increased in CIV-infected cells. Following transfection with siRNA specific to TLR3 (siTLR3) or RIG-I (siRIG-I), the mRNA expression levels of IFN-β significantly (*p* < 0.001) decreased, and the protein expression of IFN-β also decreased in infected cells. In addition, co-transfection with both siTLR3 and siRIG-I significantly reduced IRF3 (*p* < 0.001) and IFN-β (*p* < 0.001) mRNA levels. Moreover, the protein concentration of IFN-β was significantly (*p* < 0.01) lower in cells co-transfected with both siTLR3 and siRIG-I than in cells transfected with either siTLR3 or siRIGI alone. Also, the antiviral protein MX1 was only expressed in KU-CBE cells infected with CIV or treated with IFN-β or IFN-α. Thus, we speculate that IFN-β further induces MX1 expression, which might suppress CIV replication. Taken together, these data indicate that TLR3 and RIG-I synergistically induce IFN-β expression via the activation of IRF3, and the produced IFN-β further induces the production of MX1, which would suppress CIV replication in CIV-infected cells.

## Introduction

Outbreaks of canine influenza have mostly been caused by two subtypes of the canine influenza virus (CIV), H3N8 and H3N2. The equine-origin H3N8 subtype was only identified in greyhound dogs in the USA in 2004 [1], whereas the avian-origin H3N2 CIV was identified in dogs from the USA and several Asian countries, including South Korea [2-5]. H3N2 CIV can cause acute respiratory distress and severe morbidity [2, 6] and use epithelial cells in the canine upper and lower respiratory tract as hosts [2].

Influenza virus induces the production of type I interferons (IFNs) in host cells via the recognition of pathogen-associated molecular patterns, such as viral single-stranded RNA (ssRNA) or double-stranded RNA (dsRNA), by pattern recognition receptors (PRRs) in host cells [7-9]. The recognition of dsRNA, an intermediate of virus replication, is mediated by either the endosomal toll-like receptor 3 (TLR3) or cytoplasmic retinoic acid-inducible gene I (RIG-I) and melanoma differentiation-associated protein 5 (MDA5) [8, 9]. The recognition of ssRNA is mediated by endosomal toll-like receptors 7 and 8 (TLR7 and TLR8), which initiates a signaling cascade that leads to the expression of type I IFNs [7]. The TLR3-mediated signaling cascade initially activates the TIR-domain-containing adapter-inducing interferon-β (TRIF) and eventually activates interferon regulatory factor 3 (IRF3)[10]. After the recognition of viral dsRNA, RIG-I or MDA5 associates with the IFN-β promoter stimulator 1 (IPS-1) in the mitochondria, initiating a signaling cascade that leads to the activation of IRF3 or IRF7 [11]. All of these pathways finally result in the production of type I IFNs that subsequently induce the synthesis of antiviral proteins, such as orthomyxovirus- resistance GTPases (MX) and 2′-5′-oligoadenylate synthetases (OAS) [12, 13].

Although the signaling pathways for type-I IFN induction are well known for human and avian influenza viruses [14], those for CIV infection remain unclear. In a previous study, we developed a canine respiratory epithelial cell line, named KU-CBE, to study innate immune responses for H3N2 CIV infection [15]. CIV-infected KU-CBE cells demonstrated induction of IFN-β and immune responses that were different from those in CIV-infected Madin–Darby canine kidney (MDCK) cells. As a serial study, we designed experiments to determine the signaling pathways that lead to the production of type I IFNs in H3N2 CIV-infected KU-CBE cells. To the best of our knowledge, this is the first study to elucidate activation signals for type-I IFN induction during H3N2 CIV infection and the subsequent production of antiviral proteins in infected KU-CBE cells.

## Materials and Methods

### Cell Culture and Virus

KU-CBE cells were cultured in minimum essential medium (Gibco, USA) supplemented with 10% fetal bovine serum (Gibco) and antibiotics (100 U/ml penicillin and 100 g/ml streptomycin) at 37°C in a 5% CO_2_ incubator. An H3N2 canine influenza virus, (A/canine/Korea/LBM412/2008(H3N2), isolated from a dog showing signs of mild respiratory distress, was used to infect the KU-CBE cells [16].

### Gene Knockdown by siRNA

siRNAs specific to canine TLR3, RIG-I, and the non-specific control siRNA, were designed and purchased from Sigma-Aldrich (USA). These siRNAs were transfected to KU-CBE cells cultured on a 6-well plate (6 × 10^5^/well) using the Lipofectamine RNAiMAX Reagent (Invitrogen, USA) according to the manufacturer’s instructions. On the day after transfection, cells were either infected with H3N2 CIV at a 0.1 multiplicity of infection (MOI) in the presence of 1 g/ml TPCK-treated trypsin (Sigma-Aldrich), or stimulated with 1 g/ml of poly I:C (Sigma-Aldrich) for 18 h, and then used for subsequent analysis.

### RNA Extraction and Quantitative Real-Time PCR

Total RNAs were extracted using the RNeasy Mini Kit (Qiagen, USA) according to the manufacturer’s instructions. Expression of the TLR3, TLR7, TLR8, RIG-I, and MDA5 mRNAs was determined by RT-qPCR using the PrimeScript RT Reagent Kit (Takara, Japan) according to the manufacturer’s instructions (primer and probe sequences listed in Table 1). Expression levels of the IRF3, IRF7, and IFN-β mRNAs were estimated by RT-qPCR using One-Step SYBR Green Master Mix II (Takara) according to the manufacturer’s instructions (primer sequences listed in Table 2). The relative changes in mRNA expression levels of H3N2 CIV-infected KU-CBE cells to those of uninfected control cells were determined at various time points using the comparative 2^-ΔΔCt^ method [20].

### Cell Growth Curve and Cytopathic Effect (CPE)

The growth curve of KU-CBE cells was measured to determine their doubling time. Cells in the number of 2 × 105 cells/well were cultured in a 6-well plate and then harvested after 12, 24, 36, 48, and 60 h using trypsin-EDTA (Gibco). Cells were counted using a hemocytometer after trypan blue treatment (Gibco). CPEs were observed in the three groups of cells on days 1 and 3 post infection (dpi): 1) cells transfected with both siTLR3 and siRIG-I and subsequently infected with H3N2 CIV, 2) cells infected with the virus only, and 3) uninfected control cells.

### Western Blot Analysis

Cell culture supernatants were collected and cells were lysed using the RIPA buffer (Sigma-Aldrich). Cell lysates or supernatants were then run on 4–20% precast gradient gels (Koma Biotech, Korea), and all proteins were transferred onto nitrocellulose transfer membranes (GE Healthcare, UK). These were blocked overnight in phosphate-buffered saline (PBS) containing 5% skim milk at 4°C. After three washes with a washing solution containing 0.05% Tween-20 in TBS (TBS-T), the membranes were incubated with the primary antibody in TBS-T for 1 h at room temperature (20-22°C). The following monoclonal or polyclonal antibodies were used to probe the blots: mouse anti-TLR3 antibody (Abcam, USA), mouse anti-GAPDH antibody (Abcam), mouse anti-RIG-I antibody (Santa Cruz Biotechnology, USA), rabbit anti-IRF3 antibody (Sigma-Aldrich), rabbit anti-IRF7 antibody (Sigma-Aldrich), and rabbit anti-IFN-β antibody (Cloud-Clone Corp, USA). After three washes, the membranes were incubated with horseradish peroxidase-conjugated goat anti-rabbit and mouse IgG antibodies (Thermo Fisher Scientific, USA) for 1 h at room temperature. Finally, protein bands were visualized using the ECL Plus western blotting detection reagents (Amersham Biosciences, UK).

### Determination of IFN-β Concentrations

Cell culture supernatants were centrifuged at 2,300 ×*g* for 5 min to eliminate cell debris. Enzyme-linked immunosorbent assay (ELISA) kits (USCN Life Science, China) for canine IFN-β were used to determine the IFN-β protein concentrations in cell culture supernatants. The ELISA was performed according to the manufacturer’s instructions.

### Detection of Antiviral Proteins

KU-CBE cells were infected with H3N2 CIV at 0.1 MOI or treated with 1 g/ml of canine IFN-β or IFN-α (Cloud-Clone Corp, USA) for 24 h. After cells were lysed using the RIPA buffer (Sigma-Aldrich), the MX1 and OAS1 proteins were detected in cell lysates using western blots as described above. The rabbit anti-OAS1 antibody (Abcam) and rabbit anti-MX1 antibody (Abcam) were used as primary antibodies.

### Statistical Analyses

The data are expressed as the mean ± SD. The statistical analysis of the expression levels of the TLR3, TLR7, and TLR8 mRNAs was performed by one-way ANOVA using Tukey’s multiple-comparisons test as the post hoc test. The statistical analysis for the expression levels of the RIG-I, MDA5, IRF3, and IFN-β mRNAs, as well as the IFN-β protein, were performed using a t-test for paired means. A *p*-value of < 0.05 was considered statistically significant.

## Results

### Upregulation of TLR3 and RIG-I mRNA Expression in H3N2 CIV-Infected KU-CBE Cells

**Fig. 1:**
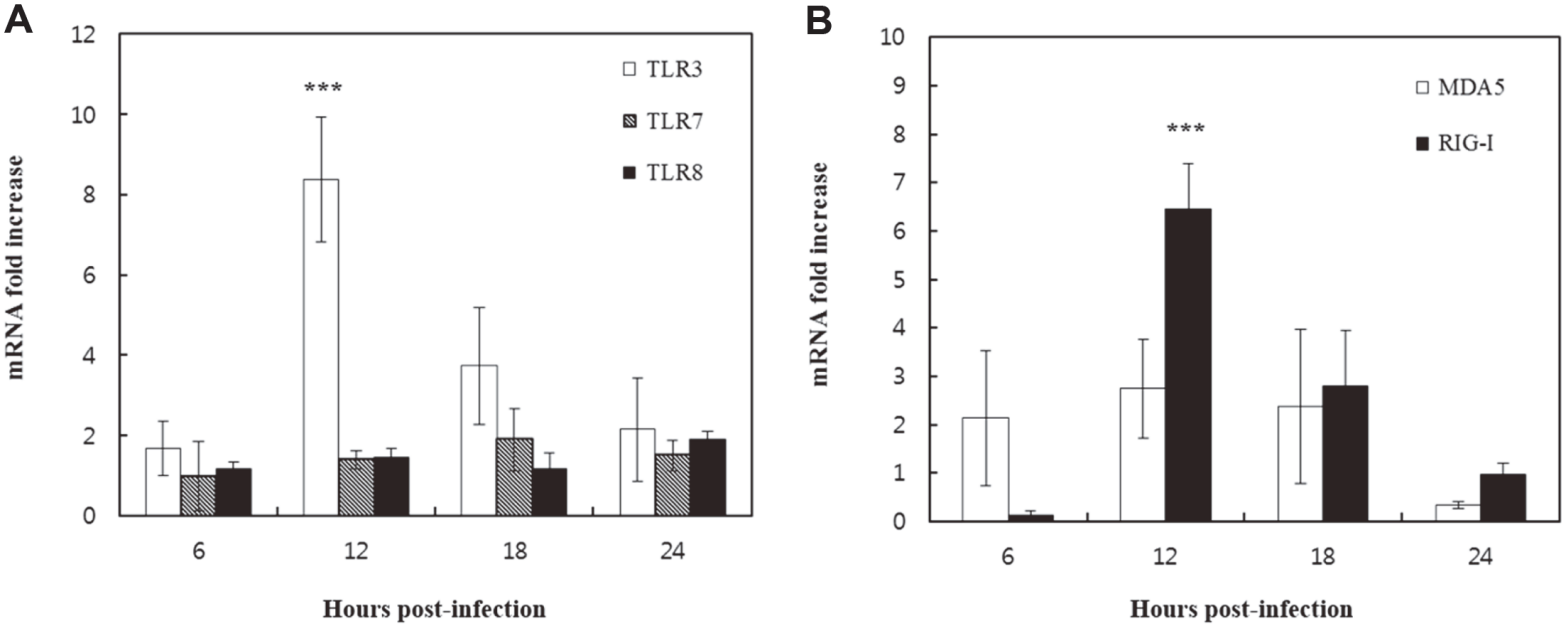
Gene expression levels of pattern recognition receptors (PRRs) in H3N2 canine influenza virus (CIV)-infected cells. (**A**) Expression of TLR3, TLR7, and TLR8. (**B**) MDA5 and RIG-I mRNA levels were assessed by RTqPCR. The expression levels of the PRRs were normalized to the constitutively expressed *GAPDH* gene. Data are expressed as the mean ± SD from triplicate experiments. ***p < 0.001.

Gene expression levels of TLR3, TLR7, TLR8, RIG-I, and MDA5 in KU-CBE cells were determined by RT-qPCR at various time points during 24 h after H3N2 CIV infection. TLR3 and RIG-I mRNA expression levels in H3N2 CIV-infected cells were significantly higher (8.4- and 6.4-fold, respectively; *p* < 0.001) than those in mock-infected cells at 12 h post-infection (Figs. 1A and 1B). However, the mRNA expression levels of TLR7, TLR8, and MDA5 in the H3N2 CIV-infected cells were not significantly increased during the experiment (Figs. 1A and 1B).

### Cytopathic Effects of Virus and Specificity of siRNAs

**Fig. 2:**
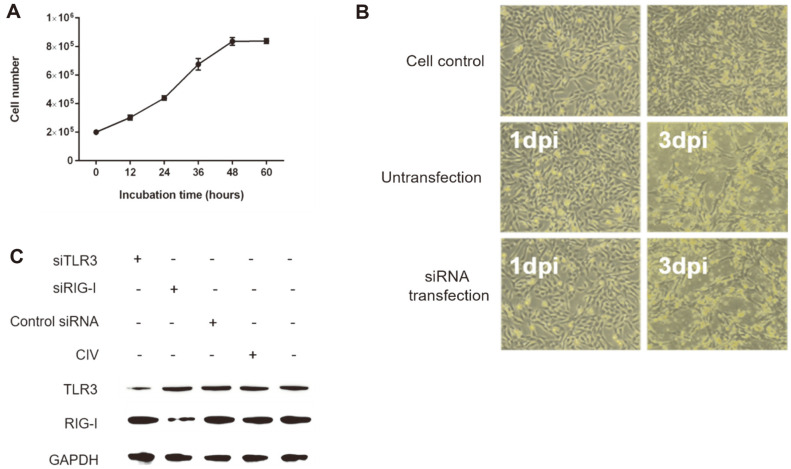
Identification of cytopathic effects and specificity of siRNAs. (**A**) Doubling times of KU-CBE cells were determined by the growth curve. The experiments were performed in triplicate. (**B**) CPE was observed in the H3N2 CIVinfected KU-CBE cells with or without siRNA treatments. (**C**) The specificity of siTLR3 and siRIG-I was identified in protein expression.

Doubling time of KU-CBE cells was determined by cell growth curve and was estimated to be about 24.5 h (Fig. 2A). CPEs were not observed in the mock-infected control cells as expected (Fig. 2B). However, CPEs were clearly identified in the cells infected with the H3N2 CIV without siRNA, as well as those transfected with both siRNA and subsequently infected with the virus (Fig. 2B). These results demonstrate that the transfection of siRNA did not affect the production of CPEs. In addition, the specificities of both siTLR3 and siRIG-I were examined. Transfection of siTLR3 specifically reduced the expression of TLR3 but did not affect the expression of RIG-I. Similarly, transfection of siRIG-I specifically decreased the expression of RIG-I but did not influence the TLR3 expression (Fig. 2C).

### Induction of IFN-β through TLR3 and RIG-I-Mediated Signaling in H3N2 CIV-Infected KU-CBE Cells

**Fig. 3:**
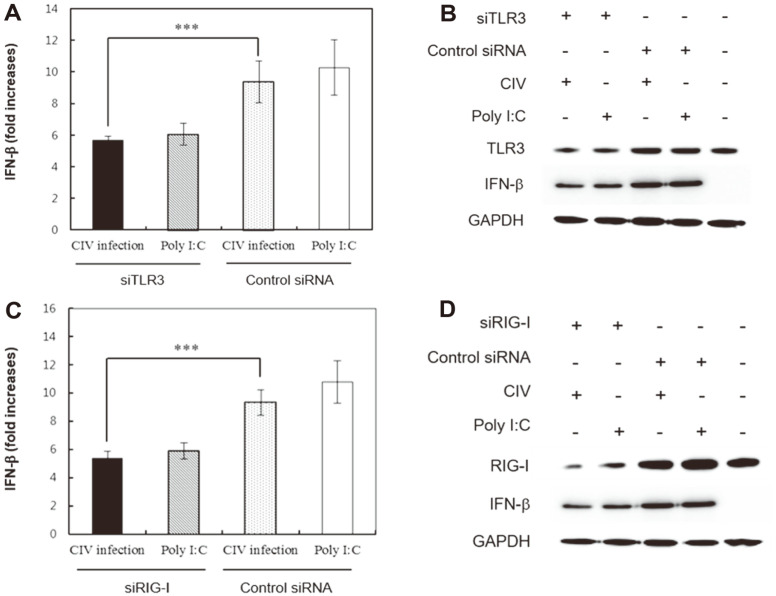
Effects of siTLR3 and siRIG-I on the IFN-β induction signaling pathway of H3N2 CIV-infected cells. (**A**) IFN-β mRNA level was significantly decreased at 18 h post-infection in siTLR3-transfected cells. (**B**) TLR3 and IFN-β protein expression levels decreased at 18 h post-infection in siTLR3-transfected cells. (**C**) The IFN-β mRNA levels significantly decreased at 18 h post-infection in siRIG-I–transfected cells. (**D**) RIG-I and IFN-β protein expression levels strongly decreased at 18 h post-infection in the siRIG-I–transfected cells. Data are expressed as the mean ± SD from triplicate experiments. ***p < 0.001.

Because both the TLR3 and RIG-I mRNA levels increased in H3N2 CIV-infected KU-CBE cells, siTLR3 was used to block the TLR3-mediated signaling pathway that leads to the induction of type-I IFNs. The mRNA levels of IFN-β were significantly decreased (*p* < 0.001) in H3N2 CIV-infected cells after transfection with siTLR3 (Fig. 3A). Given the siTLR3-mediated reduction of IFN-β mRNA expression, the expression levels of proteins in the IFN-β induction pathway were further examined under the same conditions. Notably, the production of TLR3 and IFN-β after H3N2 CIV infection was lower in the siTLR3-transfected cells than in cells transfected with control siRNA (Fig. 3B). These results indicated that recognition of the H3N2 CIV dsRNA by TLR3 leads to the production of IFN-β in H3N2 CIV-infected KU-CBE cells. In addition, siRIG-1 was used to interfere with the RIG-I–mediated signaling pathway that leads to IFN-β induction. The IFN-β mRNA levels significantly decreased (*p* < 0.001) in siRIG-I–transfected cells after H3N2 CIV infection (Fig. 3C). The protein expression of IFN-β was also examined in siRIG-I–transfected cells. The RIG-I and IFN-β protein expression levels in siRIG-I–transfected cells were strongly reduced compared with those in transfected cells with control siRNA after H3N2 CIV infection (Fig. 3D). These results verified that recognition of the H3N2 CIV dsRNA in the cytoplasm of KU-CBE cells by RIG-I activates another signaling pathway that leads to the production of IFN-β.

### Synergistic Induction of IFN-β by TLR3- and RIG-I–Mediated Signaling Pathways in H3N2 CIV-Infected KU-CBE Cells

**Fig. 4:**
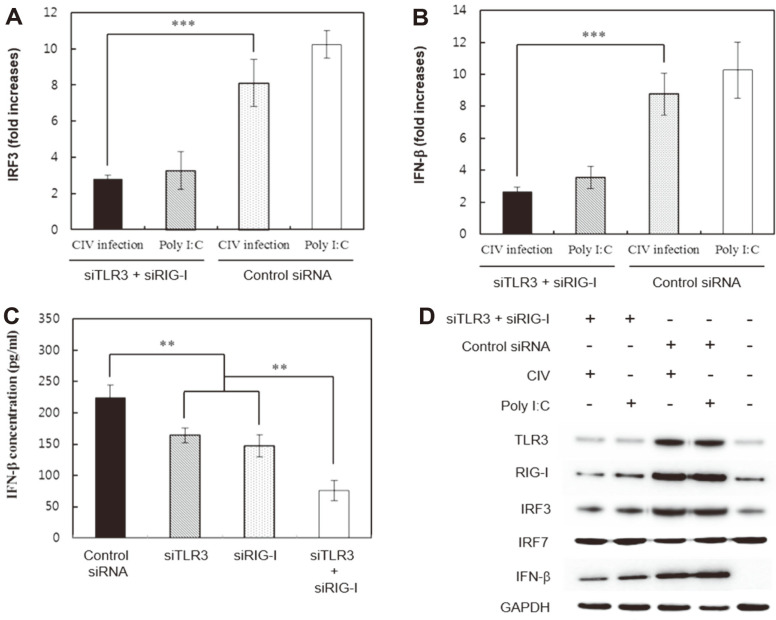
siTLR3 and siRIG-I effects on the IFN-β induction pathway of H3N2 canine influenza virus (CIV)-infected cells. (**A**) IRF3 mRNA levels significantly decreased at 18 h post-infection in cells co-transfected with both siTLR3 and siRIG-I. (**B**) IFN-β mRNA expression levels significantly decreased at 18 h post-infection in cells co-transfected with both siTLR3 and siRIG-I. (**C**) IFN-β protein production significantly decreased at 18 h post-infection in cells co-transfected with both siTLR3 and siRIG-I. (**D**) TLR3, RIG-I, IRF3, and IFN-β protein expression levels decreased at 18 h post-infection in cells co-transfected with both siTLR3 and siRIG-I. Data are expressed as the mean ± SD from triplicate experiments. **p < 0.01; ***p < 0.001.

As determined from the results of previous studies, the signaling initiated by the H3N2 CIV dsRNA-recognizing TLR3 or RIG-I induces IFN-β production. Therefore, both siTLR3 and siRIG-I were employed to examine the synergism of these two signals for IFN-β induction in H3N2 CIV-infected cells. In cells transfected with control siRNA, the mean IRF3 and IFN-β mRNA levels in H3N2 CIV-infected cells were 8.1- and 8.7-fold higher, respectively, than those in mock-infected cells (Figs. 4A and 4B). In cells co-transfected with both siTLR3 and siRIG-I, the mean IRF3 and IFN-β mRNA levels in H3N2 CIV-infected cells were 2.7- and 2.6-fold higher, respectively, than those in mock-infected cells at 18 h post-infection (Figs. 4A and 4B). As the above results show, co-transfection of both siTLR3 and siRIG-I resulted in a significant decrease (*p* < 0.001) of IRF3 and IFN-β mRNA levels in infected cells than those in control siRNA-transfected cells (Figs. 4A and 4B). Similarly, IFN-β protein synthesis in the siTLR3- and siRIG-I-co-transfected cells was significantly lower (*p* < 0.01) than that in cells transfected with either siTLR3 or siRIG-I alone after H3N2 CIV infection (Fig. 4C).

The protein expression levels of IRF3 and IFN-β in the cells transfected with the two siRNAs and infected with H3N2 CIV were lower than those in the cells transfected with the control siRNA and infected with the virus (Fig. 4D). In the case of IRF7, changes in expression of its protein by siTLR3 and siRIG-I co-transfection could not be observed (Fig. 4D). These results demonstrated that the signals initiated by both TLR3 and RIG-I synergistically activated the production of IFN-β in H3N2 CIV-infected cells.

### Detection of Antiviral Proteins in H3N2 CIV-Infected KU-CBE Cells

**Fig. 5:**
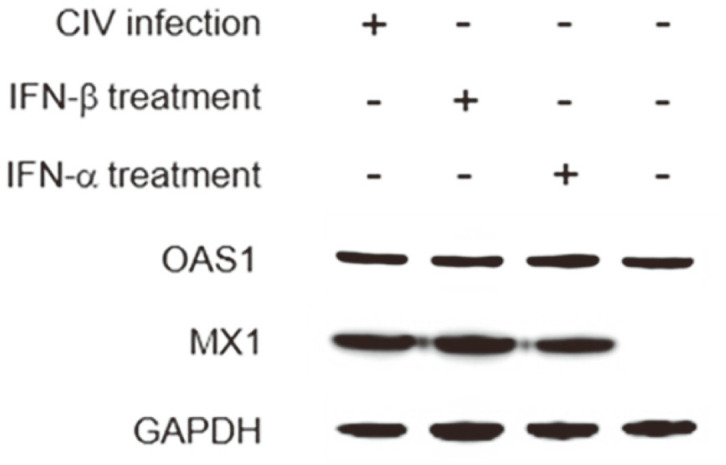
Expression of OAS1 and MX1 proteins in H3N2 canine influenza virus (CIV)-infected KU-CBE cells. The KU-CBE cells were infected with CIV or stimulated with IFN-β or IFN-α protein during 24 h. The MX1 protein detected in CIV-infected or type I IFN-treated cells.

Proteins showing antiviral effects, such as OAS1 and MX1, are known to be induced by type I IFNs in viral host cells. We identified the production of antiviral proteins in H3N2 CIV-infected KU-CBE cells. The MX1 protein was strongly expressed in KU-CBE cells infected with H3N2 CIV or treated with IFN-β or IFN-α as a positive control (Fig. 5). However, the protein production of OAS1 in H3N2 CIV-infected cells did not show obvious differences when compared with that of uninfected control cells (Fig. 5). These results indicated that MX1 protein expression only resulted from H3N2 CIV infection in KU-CBE cells.

## Discussion

In a previous study, we showed that KU-CBE cells abundantly express IFN-β after H3N2 CIV infection [15]. In this study, we aimed to investigate the type I IFN pathway and its production of antiviral proteins in KU-CBE cells infected with the H3N2 strain of CIV. TLR3 recognizes the dsRNA intermediate in the endosome during replication of the influenza virus. According to a previous study, TLR3 expression in pulmonary epithelial cells was upregulated by human influenza virus and by dsRNA [21]. Similarly, this study demonstrated that the TLR3 mRNA levels increase in KU-CBE cells after H3N2 CIV infection. When the TLR3 expression was blocked by siTLR3 in H3N2 CIV-infected KU-CBE cells, a reduction in the IFN-β synthesis was observed. These results suggest that the recognition of H3N2 CIV dsRNA by TLR3 is an essential initial step for the expression of type I IFNs in host cells.

TLR7 acts as a receptor for the ssRNA influenza virus and plays an important role in the synthesis of type I IFNs in plasmacytoid dendritic cells [22]; however, TLR7 might not induce the production of type I IFNs in other types of cells [23]. Similarly, a type I IFN production mediated by TLR7 was not expected in our study because the H3N2 CIV infection did not highly induce the expression of TLR7 mRNA levels in KU-CBE cells.

Several other studies have shown that RIG-I also activates the expression of type I IFNs by recognizing the dsRNA of the influenza virus [24, 25]. In this study, increased levels of RIG-I expression were identified in H3N2 CIV-infected KU-CBE cells, and when RIG-I expression was blocked by siRIG-I, IFN-β production was reduced. These results suggest that both TLR3 and RIG-I are related to type I IFN expression in KU-CBE cells when infected with H3N2 CIV.

Different PRRs can cooperatively induce type I IFNs in virally infected cells. The inhibition of intracellular RNA virus sensors TLR3, RIG-I, and MDA5 via their specific siRNAs reduced the mRNA expression of innate cytokines, including IFN-β in bronchial epithelial cells infected with rhinovirus [26]. These sensors were also activated upon dengue virus infection in a human hepatoma cell line, and their activation synergistically resulted in higher expression levels of type I IFNs [27]. In this study, we also demonstrated the synergistic effect of TLR3 and RIG-I on IFN-β production in H3N2 CIV-infected KU-CBE cells through IRF3-involved signaling. IFN-β synthesis was more reduced in cells co-transfected with siTLR3 and siRIG-I than in cells transfected with either siTLR3 or siRIG-I alone. Similar to our results, it was recently reported that signaling through both RIG-I and TLR3 is important for inducing type I IFNs in primary human alveolar epithelial cells infected with influenza virus [28]. These results support that at least two signaling pathways initiated by TLR3 and RIG-I are required for the induction of higher IFN-β levels in canine and human respiratory epithelial cells infected with influenza virus.

Type I IFNs produced by viral infections bind to IFN-β/α receptors and induce the expression of several IFN-stimulated proteins, such as MX and OAS [29]. As expected, our data showed that H3N2 CIV infection only results in the production of MX1 protein in KU-CBE cells. A previous study showed that lung adenocarcinoma (A549) cells constitutively expressed OAS1 and OAS3 mRNA [30]. Therefore, it is not surprising that the OAS1 protein was produced basally in uninfected KU-CBE cells.

In conclusion, our results indicate that TLR3 and RIG-I synergistically induce IFN-β expression through the activation of IRF3 in KU-CBE cells infected with H3N2 CIV. The infected cells eventually produce MX1, known as antiviral effector proteins. These results can contribute to a more thorough understanding of the IFN-β induction signaling pathway of the innate immune response in the canine respiratory tract after H3N2 CIV infection. Moreover, KU-CBE cells may be utilized to study the host defense innate immune mechanism for H3N2 CIV infection.

## Figures and Tables

**Table 1:** DNA sequences of primers and probes used for TaqMan RT-qPCR.

Gene	Primer and probe	Sequence (5′–3′)	Product length (bp)	GenBank accession number	Reference
GAPDH	Forward	TGAACGGATTTGGCCGTATTGG	90	NM001003142	[17]
	Reverse	TGAAGGGGTCATTGATGGCG			
	Probe	CAGGGCTGCTTTTAACT			
TLR3	Forward	GCAACACCCAGCTACACACA	130	XM540020.2	
	Reverse	ATGTGGAAGCCAGACAAAGG			
	Probe	TCACCATGCTCGATCTTTCCCACA			
TLR7	Forward	GCCCTTTTTCTGATGGTGATT	100	DQ333224.1	
	Reverse	CGCCGATACCCCTTTATTTT			
	Probe	CCACCTCTACTTCTGGGACGTGTGG			
TLR8	Forward	TGATCTGCCCGAGTTAGAAG	135	JF681168.1	
	Reverse	ATGCTGTTGTGGCTCAAGTT			
	Probe	TCCTAGGCGGTGCGTCACCC			
MDA5	Forward	AACAGGCAAACTTGCTTTG	121	XM545493.4	This study
	Reverse	GCCCTCTTTCTCTGGACTTG			
	Probe	ATCACAAGAAGATGGCCCTG			
RIG-I	Forward	CTTGTTCTGCATAGGAGCCA	158	XM003639385.1	This study
	Reverse	TGAACAAGGGATAAATGGCA			
	Probe	CCTGTTTACCAATGCAGCAA			

**Table 2:** DNA sequences of primers used for SYBR Green RT-qPCR.

Gene	Primer	Sequence (5′–3′)	Product length (bp)	GenBank accession number	Reference
*IRF3*	Forward	GTGATGCTCAAGGTTGTTCC	113	XM005616307	This study
	Reverse	GGGTAGCTGTTGGAAATGTG			
*IFN-β*	Forward	CCAGTTCCAGAAGGAGGACA	200	NM001135787	[18]
	Reverse	TGTCCCAGGTGAAGTTTTCC			
*β-actin*	Forward	CCGCGAGAAGATGACCCAGA	81	Z70044	[19]
	Reverse	GTGAGGATCTTCATGAGGTAGTCGG			
